# Halloysite nanotube-based electrospun ceramic nanofibre mat: a novel support for zeolite membranes

**DOI:** 10.1098/rsos.160552

**Published:** 2016-12-21

**Authors:** Zhuwen Chen, Jiaying Zeng, Dong Lv, Jinqiang Gao, Jian Zhang, Shan Bai, Ruili Li, Mei Hong, Jingshen Wu

**Affiliations:** 1 Guangdong Provincial Key Laboratory of Nano-Micro Materials Research, School of Chemical Biology and Biotechnology, Peking University Shenzhen Graduate School, Shenzhen 518055, People's Republic of China; 2 Department of Mechanical and Aerospace Engineering, The Hong Kong University of Science and Technology, Clear Water Bay, Hong Kong SAR, People's Republic of China; 3 Shenzhen Engineering Laboratory for Water Desalinization with Renewable Energy, School of Environment and Energy, Peking University Shenzhen Graduate School, Shenzhen 518055, People's Republic of China

**Keywords:** halloysite, electrospin, nanofibre, zeolite, ceramic membrane

## Abstract

Some key parameters of supports such as porosity, pore shape and size are of great importance for fabrication and performance of zeolite membranes. In this study, we fabricated millimetre-thick, self-standing electrospun ceramic nanofibre mats and employed them as a novel support for zeolite membranes. The nanofibre mats were prepared by electrospinning a halloysite nanotubes/polyvinyl pyrrolidone composite followed by a programmed sintering process. The interwoven nanofibre mats possess up to 80% porosity, narrow pore size distribution, low pore tortuosity and highly interconnected pore structure. Compared with the commercial α-Al_2_O_3_ supports prepared by powder compaction and sintering, the halloysite nanotube-based mats (HNMs) show higher flux, better adsorption of zeolite seeds, adhesion of zeolite membranes and lower Al leaching. Four types of zeolite membranes supported on HNMs have been successfully synthesized with either *in situ* crystallization or a secondary growth method, demonstrating good universality of HNMs for supporting zeolite membranes.

## 1. Introduction

Zeolites are polyporous molecular sieve materials consisting of an aluminosilicate tetrahedron framework. Considering all zeolite framework types, the Si/Al ratio can be extended from one to infinity, which shows significant impacts on their hydrophilicity, acid stability and ion exchange capacity. Their properties can be further tuned by substituting framework atoms with heteroatoms such as phosphorus, titanium, boron and others. Recently, inorganic zeolite membranes have attracted more attention as promising candidates for energy-efficient and economic separation application [1–4], as well as for sensing [5] and reactor [6–8] applications. Among over 200 types of zeolite framework types, less than 20 have been fabricated into effective membranes because of the defects in the zeolite membrane [9]. The low quality of supports is one of the major factors that limits the mass application of zeolite membranes [10].

Generally, zeolite membranes are supported by macroporous substrate such as alumina [11–16], mullite [17], stainless steel [18,19] or glass [20] to strengthen the whole structure and minimize zeolite film thickness. This asymmetric membrane structure creates gradient pore channels along the molecule diffusion pathway and reduces transport resistance, and thus provides higher flux than unsupported ones. Therefore, an appropriate support is of vital importance in zeolite membrane performance [11]. The flux resistance of a zeolite membrane is largely located in the support layer [21], and the pervaporation flux has been reported to depend almost linearly on the support porosity [22]. For industrial application of zeolite membranes, a larger increase in flux is required [10] and thus the support porosity has to be maximized. Besides accounting for a large portion of zeolite membrane performance, support qualities such as pore connectivity also have a profound effect on the success of zeolite membrane fabrication. Unmatched support morphology with the growing zeolite membranes leads to crack formation [23] and inferior thermal stability [24]. The seeding–secondary growth method is by far the most widely employed method for zeolite membrane synthesis [12] in order to control zeolite nucleation and crystal growth on the support surface, and decrease intercrystalline defects. However, the incompatibility between zeolite seeds and as-used support still remains a limitation. Features of the support, such as pore size distribution, porosity, surface curvature and surface roughness could determine the degree and strength of zeolite seed attachment to the porous support.

The most commonly used support for zeolite membrane synthesis is the α-Al_2_O_3_ support manufactured via consolidation of bulk alumina powders. The pores of the support are generated by the interparticle voids, whereas the bulk powder of α-Al_2_O_3_ acting as space filler typically results in low porosity [25]. During a seeding process, the zeolite seeds fall easily from the smooth surface of the bulk α-Al_2_O_3_ grains owing to low adhesion, causing inhomogeneous seed distribution on the support. To deal with the incompatibility, molecular linkers such as 3-halopropylsilyl reagents [26,27] have been used to modify the support and/or zeolite seed surface, but this modification process is labour-intensive. To overcome these disadvantages, in this study, we employed a novel electrospun ceramic membrane featuring nanofibrous morphology as support for zeolite membrane fabrication. Electrospinning technique is widely employed as the simplest technique to fabricate nanostructure membranes, which can produce continuous micro- or nanofibres [28]. The nanofibrous membranes are particularly suitable to be a supporting material owing to their high effective porosity, large surface area and highly interconnected pore structure. Very few self-standing electrospun ceramic membranes have been reported owing to the complicated and rigorous preparation technology. Fibrous γ-alumina [29], SiO_2_, SiO_2_–TiO_2_ composite [30–32], ZrO_2_ [33] and Pd/CeO_2_–TiO_2_ membranes [34] have been fabricated by the electrospinning technique, for use as particulate filtration media with low pressure drop, for water filtration, surface functionalization or further catalytic application. The thicknesses of these membranes were all in the micrometre range. When used in zeolite membranes, the desirable supports should be stable and self-standing with a thickness in the millimetre scale. Nanotube material-based membranes have been reported to allow high flux [35,36] and would be suitable to act as support. In this study, halloysite nanotubes (HNTs) have been chosen as the ceramic support material owing to their high mechanical strength and abundance in nature [37] compared with other nanotube materials. After properly selecting the precursor and adjusting the electrospinning parameters, we firstly fabricated a well-interwoven HNTs/PVP nanofibre mat by the electrospinning process. After controlled sintering, a series of HNT-based ceramic nanofibre mats (HNMs) have been obtained. The flat self-standing HNMs had a thickness up to 5 mm, which is an order of magnitude thicker than those currently attainable for fibrous ceramic membranes. It has a three-dimensional highly interpenetrated pore network that presents up to 80% porosity, narrow pore size distribution and low pore tortuosity. On these novel supports with high adaptability, four representative types of zeolite membranes have been synthesized. The selection of the zeolite framework types was intended to cover the entire zeolite composition range, including lowest silica hydrophilic NaA, pure-silica hydrophobic silicalite-1, NaY with a Si/Al ratio of approximately 2 and heteroatoms-substituted AlPO_4_-5. All these membranes synthesized were thin and defect-free, demonstrating the great potential of the high-porosity, low-cost nanofibre mat as a possible support for all-zeolite-type membranes.

## 2. Experimental section

### 2.1. Materials

All reagents used in this study were purchased from commercially available sources without further purification, unless specified otherwise. Sodium aluminate (powder, greater than or equal to 99%), sodium hydroxide (sheet, greater than or equal to 99%), aluminium isopropoxide (powder, greater than or equal to 99%), tetrapropylammonium hydroxide (TPAOH, 40% w/w aqueous solution) and triethylamine (TEA) were purchased from Adamas, HNTs from Imerys Tableware New Zealand Limited, tetraethyl orthosilicate (TEOS, greater than or equal to 99%) from Aladdin, Ludox (25% aqueous solution of SiO_2_) from Qingdao Ocean Co., Ltd., H_3_PO_4_ (85 wt% aqueous solution) from Tianjin ZhiYuan Reagent Co., Ltd, polyvinyl pyrrolidone (PVP, K90, *M*_w_ = 130 000) from Beijing Biodee Biotechnology Co., Ltd. Deionized water was used throughout the experiments. Commercially available α-Al_2_O_3_ supports with a thickness of 2–3 mm from Foshan Nanhai Jingang Technology Co., Ltd. (denoted as FNJ) were used as a reference.

### 2.2. Preparation of halloysite-based electrospun nanofibre mat

HNTs were dispersed in an ethanol/water mixture with a weight ratio of 8 : 1 by ultrasonication. After 1 h, 12 wt% PVP was added and magnetically stirred until the mixture became homogeneous. HNTs/PVP nanofibre membranes were prepared using a needle-free electrospinning technology. The electrospinning system was operated with the following parameters. The distance between spinning electrode and collecting electrode was 120–240 mm. The applied voltages were in the range of 0–80.0 kV. Relative humidity was approximately 40%. An alumina foil, as the collecting electrode, was electrically grounded and covered by a layer of smooth silicon oil paper. The HNTs/PVP nanofibre membranes were cut using a steel mould with a diameter of 45 mm and consolidated to be multilayered HNTs/PVP nanofibre membranes by applying compressive pressures ranging from 4.44 to 16.7 MPa. Then, the shaped membranes were first sintered from room temperature to 1100°C in N_2_ and then further heated to 1200–1400°C in air with a 5°C min^−1^ heating rate.

### 2.3. Zeolite membrane synthesis

#### 2.3.1. NaA (LTA) zeolite membrane synthesis

The gel composition used for NaA zeolite seed crystals was Al_2_O_3_ : SiO_2_ : Na_2_O : H_2_O = 1 : 2 : 2 : 120. An aluminate solution was obtained by adding 2 g of sodium hydroxide to a solution of 4.1 g of sodium aluminate in 25 ml of water and stirring for more than 15 min. Then, the clear solution was dosed into a silicate solution including 12 g of Ludox and 20 ml of water to form an azure gel. The gel was aged for 8 h at 50°C, followed by a 3 h hydrothermal synthesis at 100°C under constant mechanical stirring. After the synthesis, the mixture was quenched by washing with deionized water until the pH of the supernatant solution was close to 7, and then the white solid (named as NaA zeolite seed crystals) was dried at 60°C. In a seeding procedure, the seeds were dispersed in a 20% ethanol–water solution before a wetting–rubbing operation to implant seeds on the HNT-based nanofibre mat (HNM) support. The seeded supports were then dried at 60°C overnight. The composition of the gel for NaA zeolite membrane secondary growth was Al_2_O_3_ : SiO_2_ : Na_2_O : H_2_O =1 : 2 : 2 : 150. The solution for zeolite membrane synthesis was obtained by adding 2 g of sodium hydroxide to the mixture of 4.1 g of sodium aluminate in 38.5 ml of water and stirred for more than 15 min. Then the clear solution was dosed into a solution including 12 g of Ludox and 20 ml of water to form an azure gel. The gel was aged for 8 h at 50°C. The secondary growth was performed in an autoclave by immersing the seeded supports in the gel and then heating to 100°C for 3.5 h followed by a water-washing step with a wet brush several times until the pH of the solution on membrane surface was close to 7. The secondary growth procedure was repeated to get a continuous NaA (LTA) membrane.

#### 2.3.2. Silicalite-1 (MFI) zeolite membrane synthesis

The molar ratio of the synthesis mixture used for silicalite-1 zeolite seed crystals was TPAOH : TEOS :H_2_O = 0.2 : 1 : 150. TEOS (5 g) was added to an aqueous solution of 2.5 g of TPAOH and 65 ml of water, which was then stirred for ageing at 50°C for 8 h. The reaction mixture was poured into an autoclave and heated at 180°C for 9 h; then the precipitate was washed with water several times until the pH of the supernatant was close to 7 and dried at 60°C. The seeding procedure was conducted by wetting–rubbing a 50 : 50 mixture of the silicalite-1 seeds suspension in water and ethanol onto the surface of an HNT-based nanofibre mat support, and then drying at 60°C. The gel composition used for a secondary hydrothermal growth of silicalite-1 membrane was TPAOH : TEOS : H_2_O = 0.17 : 1 : 165 [11]. The synthesis solution for the silicalite-1 membrane was obtained by slowly adding 6 g of TEOS to an aqueous solution of 2.5 g of TPAOH and 84 ml of water, and ageing at 50°C for 8 h. The seeded support was placed into the synthesis solution and heated in an autoclave at 180°C for 9 h. Then the membrane was washed with a wet brush and dried at 60°C overnight. The as-made membrane was calcined in a furnace at 550°C with a heating and cooling rate of 1°C min^−1^ to remove the organic templates when needed.

#### 2.3.3. NaY (FAU) zeolite membrane synthesis

The experimental processes for NaY zeolite seeds and membrane synthesis were the same as for LTA zeolite seeds and membranes except the composition, synthesis temperature and time. The composition for NaY zeolite seeds was Al_2_O_3_ : SiO_2_ : Na_2_O : H_2_O = 1 : 10 : 8 : 400 [38]; the gel was heated at 100°C for 12 h. The composition for the FAU zeolite membrane was Al_2_O_3_ : SiO_2_ : Na_2_O : H_2_O = 1 : 12.8 : 17 : 975 [39], and the gel was heated at 90°C for 15 h.

#### 2.3.4. Aluminophosphate AlPO_4_-5 (AFI) membrane synthesis [19]

Phosphoric acid solution (7.5 g) was dosed into a solution composed of 10.2 g of aluminium isopropoxide and 87.0 ml of water and stirred at room temperature for 3 h; then 2.0 g of TEA was added and stirred for another 2 days at room temperature to obtain the synthesis gel with a composition of Al_2_O_3_ : P_2_O_5_ : TEA : H_2_O = 1 : 1.3 : 1 : 200. The HNT-based nanofibre mat supports and commercially available α-Al_2_O_3_ supports were placed into the same autoclave filled with the aforementioned gel and heated at 180°C for 10 h, and then washed with deionized water and dried at 60°C overnight. The as-made membranes were calcined in a furnace at 550°C to remove the organic template.

### 2.4. Characterization

Porosity of the nanofibrous membranes was measured with Archimedes' principle, using the GB/T 1966–1996 method [40]. Powder X-ray diffraction (XRD) patterns of zeolite crystals and membranes were recorded, using a Rigaku D/Max-2200 PC diffractometer in the diffraction angle range 2*θ* = 10–70° or 2*θ* = 5–50° with Cu–Ka radiation (*λ* = 1.5418 Å) at 40 kV, 40 mA. Membrane morphology and chemical composition analyses were performed on a scanning electron microscope (SEM, JEOL JSM-7800F and JEOL JSM-6390) and energy dispersive X-ray analyser (EDS, Oxford, X-Max20 AZtec Energy 250, model no. XMX1112). The powder samples were either dispersed in water and dropped on silicon wafers which were attached onto a conductive adhesive for SEM observation, or directly mounted onto the conductive adhesives for EDS measurements. The zeolite membranes were mounted onto the conductive adhesives directly for SEM and EDS measurements. Mercury intrusion porosimetry (MIP) measurement was executed in an automatic mercury porosimeter (AutoPore IV 9500), and the water flux was tested by a home-made filtration facility. Contact angle measurements were carried out in a drop shape analyser (DSA30, KRUSS GmbH). The zeolite seed crystals were washed with water by centrifugation in a refrigerated centrifuge (Thermo Scientific-Sorvall RC 6Plus, Fiberlite F21s-8 × 50y).

## 3. Results and discussion

### 3.1. The properties of HNM supports

**Scheme 1.**
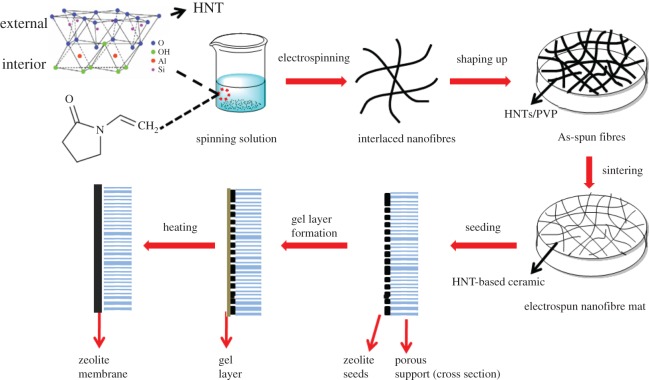
Schematic of the process for HNM support and the subsequent secondary growth method for zeolite membrane fabrication.

**Figure 1.**
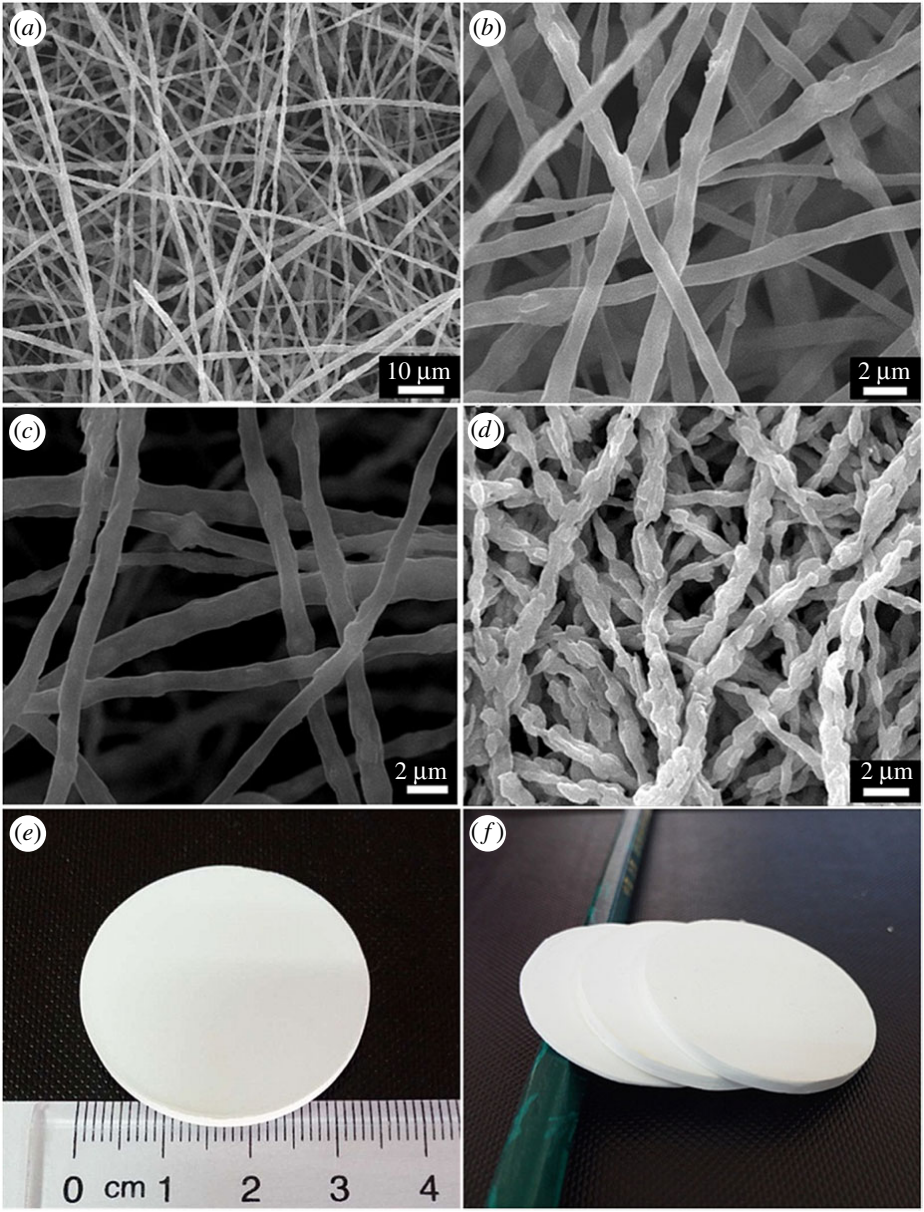
(*a*,*b*) SEM images of HNTs/PVP fibre membrane taken under different magnification before heating, (*c*) SEM image of HNTs/carbon membrane after heating at 1100°C under N_2_, (*d*) SEM image of HNMs after sintering in air at 1200°C, (*e*,*f*) appearance of HNMs.

The schematic diagram of the process for fabricating HNMs is presented in scheme 1. First, an electrospinning solution containing HNTs and PVP was sprayed to form an interlaced HNTs/PVP composite nanofibre membrane. Based on the morphology of the HNTs/PVP membrane shown in figure 1*a*,*b*, composite nanofibres possessing smooth surfaces were interwoven with each other and were consecutive and randomly oriented to the in-plane direction. The diameter of the nanofibres was uniform and in the range of 1.01 ± 0.27 µm. During the sintering process, HNTs/PVP nanofibres were subsequently converted into HNTs/carbon in N_2_ at 1100°C (figure 1*c*). Finally, bulk HNMs with a diameter up to 50 mm and thickness from 0.1 to 5 mm (figure 1*e*,*f*) were fabricated by further sintering in air above 1200°C. Based on the SEM micrograph, the pores formed by interlaced nanofibres could be clearly observed (figure 1*d*). The nanofibres' diameter was 0.83 ± 0.45 µm, a little smaller than that of the non-sintered nanofibres. The surface of nanofibres was slightly rougher, as a possible consequence of the removal of the organic polymers.

**Figure 2.**
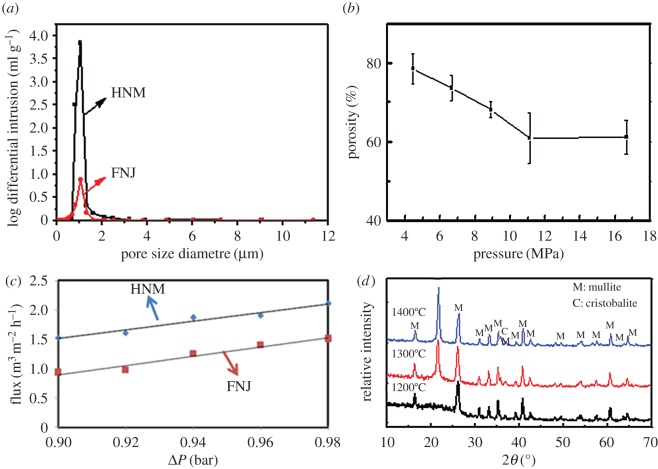
(*a*) Pore size distribution of an HNM and a commercial FNJ support, (*b*) porosities of HNM obtained under different pressing pressures before sintering, (*c*) water flux of an HNM, compared with that of a conventional FNJ support. The line is the linear regression. (*d*) XRD patterns of HNMs obtained at different sintering temperatures.

The pore size distribution of an HNM and a commercial support named FNJ was obtained by MIP measurement (figure 2*a*). The pores of the HNM were in the size range of 0.8–1.3 µm (electronic supplementary material). A sharper peak of the HNM at 1.05 µm means relatively narrower pore size distribution than the FNJ support, which could decrease defects in the subsequent step of depositing zeolite seeds. Pore structure was also characterized by MIP measurement (table 1). The total pore volume of the HNM was 0.73 ml g^−1^, which was much higher than that of the FNJ support at 0.17 ml g^−1^. The tortuosity factor of the HNM was lower than 2, suggesting the pores in the HNM are approximately cylindrical and more homogeneous [41–43]. Additionally, a lower tortuosity at 30.36 for the HNM compared with 62.18 for the FNJ conferred an almost four times higher permeability of 3.54 md for the HNM compared with 0.76 md for the FNJ support. Measured by Archimedes' method (figure 2*b*), the porosity of HNMs could be adjusted from 60.9% to 80% as the applied pressing pressure before sintering decreased from 11.1 to 4.4 MPa (electronic supplementary material), which was much higher than that of the FNJ support at 38.5%. Owing to the higher pore volume, lower tortuosity, higher permeability and higher porosity, the HNM possessed a higher water flux than the commercial α-Al_2_O_3_ support. A similar trend of almost linear increase in water flux as a function of pressure drop was observed (figure 2*c*). At a transmembrane pressure of 0.98 bar, the water flux for the HNM with a porosity of 67% was 2.1 m^3 ^m^−2 ^h^−1^, 40% higher than that for the α-Al_2_O_3_ support, at 1.5 m^−3^ m^−2^ h^−1^.

**Table 1.** Pore structure analysis of an as-synthesized HNM and a commercial FNJ support.

	pore volume (ml g^−1^)^a^	total pore area (m^2^ g^−1^)^a^	tortuosity factor^b^	tortuosity^b^	permeability (mD)^b^
HNM	0.73	2.53	1.69	30.36	3.54
FNJ	0.17	0.61	2.11	62.18	0.76

^a^Intrusion data were summarized at 3 × 10^4^ psia.

^b^Pore structure parameters were calculated at the threshold pressure.

The relationship between strength of the HNMs and sintering temperature was also investigated. HNT is a two-layered aluminosilicate whose outer surface is silica and the inner lumen is alumina (scheme 1). Based on the XRD results (figure 2*d* and electronic supplementary material), the exterior amorphous SiO_2_ transformed to mullite at 1200°C. However, the membrane was still fragile until the cristobalite phase appeared. After further sintering to 1300°C, a sharp peak appeared at 2*θ* = 21.7°, which reveals the generation of the cristobalite phase of SiO_2_. Accordingly, the membrane became stronger and the burst strength of the HNM increased to 0.58 ± 0.14 bar mm^−1^. When the sintering temperature further increased to 1400°C, the amount of cristobalite increased. As a result, the burst strength of the HNM increased to 1.78 ± 0.40 bar mm^−1^.

**Figure 3.**
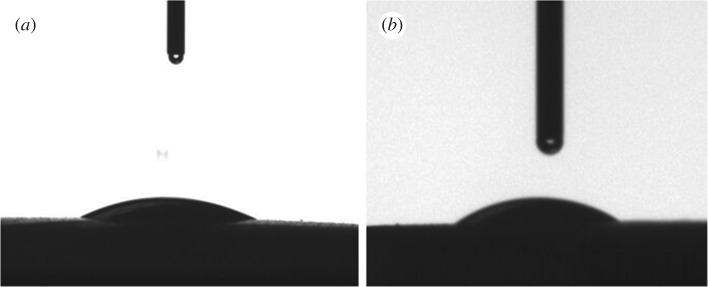
(*a*) Water contact angle and (*b*) ethanol contact angle on HNM.

The instantaneous water contact angle on the HNM was 16° (figure 3*a*) and the ethanol contact angle was 22.8° (figure 3*b*), suggesting the HNM has high affinity both to water and alcohol. This might be beneficial for zeolite crystal seeding in an alcohol/water environment, and for zeolite membrane synthesis in hydrothermal conditions.

### 3.2. Compatibility of the zeolite seeds to the HNM supports and secondary hydrothermal synthesis

**Figure 4.**
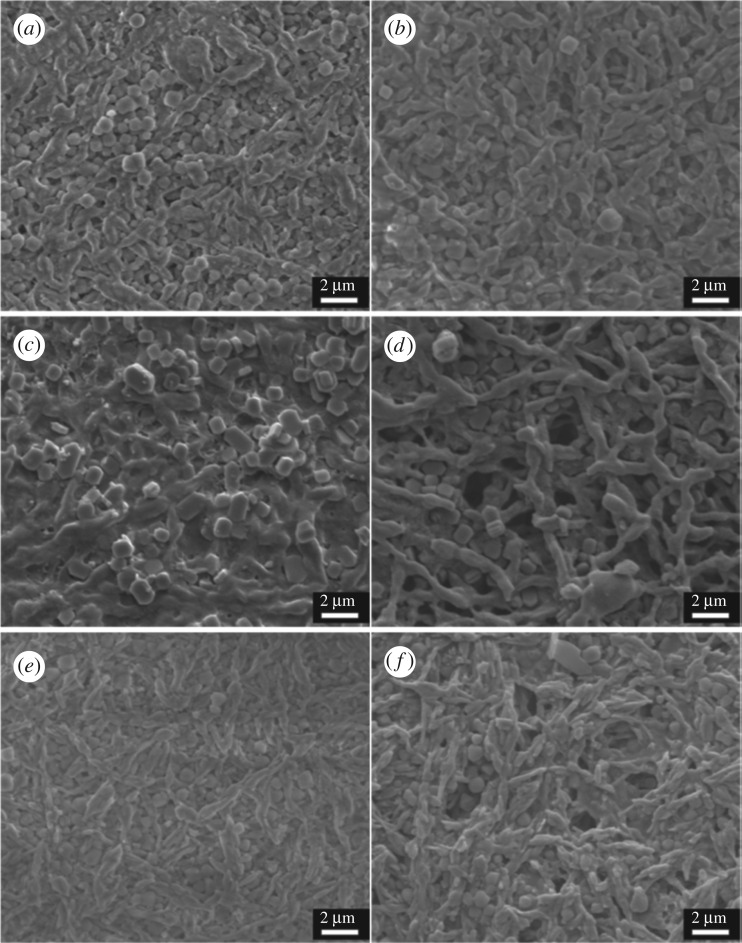
SEM images of (*a*) a NaA crystal-seeded HNM support before and (*b*) after a 2 min ultrasonic treatment; SEM images of (*c*) a silicalite-1 crystal-seeded HNM support before and (*d*) after a 2 min ultrasonic treatment; SEM images of (*e*) a NaY crystal-seeded HNM support before and (*f*) after a 2 min ultrasonic treatment.

Pre-seeding of the support offers nucleation sites for further zeolite crystal growth into a continuous zeolite layer under a typically less concentrated nutrient solution [44]. A seeding procedure can help prevent the formation of competing impure zeolite phases [44,45]. Three different kinds of seeds, including NaA with a Si/Al ratio of 1, silicalite-1 with a Si/Al ratio of infinity, and NaY with a Si/Al ratio between 1.5 and 2.5 have been successfully applied. The most hydrophilic NaA (LTA) membrane was first considered as it is the only kind of zeolite membrane commercialized for solvent dehydration [1,46–50]. The NaA zeolite seeds synthesized exhibited a cubic shape with a size range of 0.8–1.3 µm (electronic supplementary material, figure S1*a*), slightly larger than the HNM support pore size. They can be inserted into the pores uniformly and tightly on the intertwined HNT-based nanofibre support (figure 4*a*), which remained locked even after an ultrasonic treatment for 2 min (figure 4*b*). Comparatively, using commercial α-Al_2_O_3_ supports, the uneven pore size distribution led to inhomogeneous distribution of the seeds (electronic supplementary material, figure S2*a*), and some bare parts of support without seeds can be clearly seen owing to the large flat surfaces of bulk α-Al_2_O_3_ particles. After the same ultrasonic treatment, seeds can hardly be found on the support (electronic supplementary material, figure S2*b*). The uneven pore distribution caused a large amount of seed aggregation in large pores which may fall off during the secondary hydrothermal synthesis process.

To evaluate the wide adaptability of the novel halloysite-based nanofibre mat as a support for anchoring zeolite crystals, we fabricated two other types of zeolite seeds, which are pure-silica silicalite-1 zeolite with MFI topology and NaY zeolite having a silicon over aluminium ratio higher than 1.5 with FAU topology. In both cases, the seeds were well distributed on the HMM supports. The highly connected pore network intersected only by the high-aspect-ratio thin nanofibres ensured effective embedding of the seed crystals resistant to external ultrasonic forces. For silicalite-1 crystals, most of the coffin-shaped MFI seeds with a size of approximately 1.1 × 0.9 × 0.4 µm (electronic supplementary material, figure S1*c*) were inserted into the network of the support, and most of the seeds adhered to the surface tightly (figure 4*c*). After an ultrasonic treatment for 2 min, the seeds on the surface peeled off, but the ones in the holes remained (figure 4*d*). Similarly, octahedron-shaped NaY seeds with a size range of 0.8–1.3 µm (electronic supplementary material, figure S1*e*) were coated firmly on the HNM supports (figure 4*e*). As expected, most of these seeds were still firmly stuck in the pore network of the support mat and did not detach after an ultrasonic treatment for 2 min (figure 4*f*). On the contrary, on ceramic α-Al_2_O_3_ supports, seeding of silicalite-1 and NaY crystals exhibited a lower degree (electronic supplementary material, figure S2*c*,*e*) and strength after the same corresponding ultrasonic treatment (electronic supplementary material, figure S2*d*,*f*).

**Figure 5.**
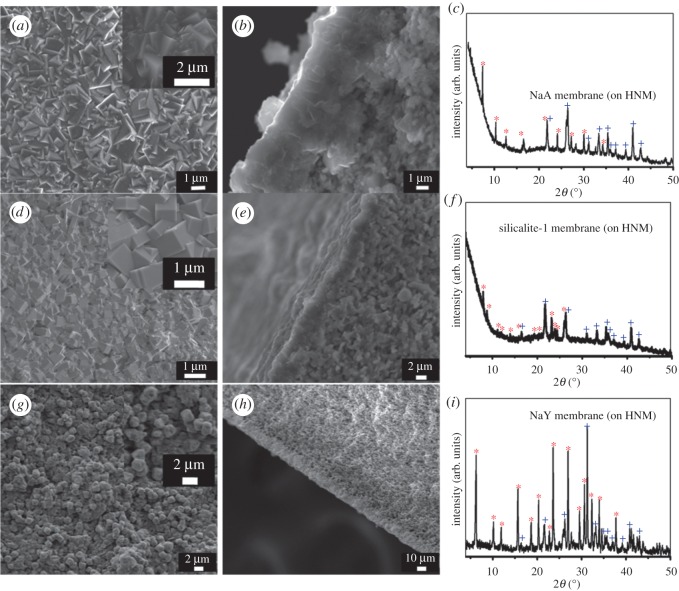
(*a*) SEM top view image, (*b*) SEM cross-sectional image and (*c*) XRD pattern of the zeolite NaA membrane; (*d*) SEM top view image, (*e*) SEM cross-sectional image and (*f*) XRD pattern of the zeolite silicalite-1 membrane; (*g*) SEM top view image, (*h*) SEM cross-sectional image and (*i*) XRD pattern of the zeolite NaY membrane. Diffraction peaks of zeolite phases were marked with asterisks, and diffraction peaks of HNMs were marked with crosses.

**Figure 6.**
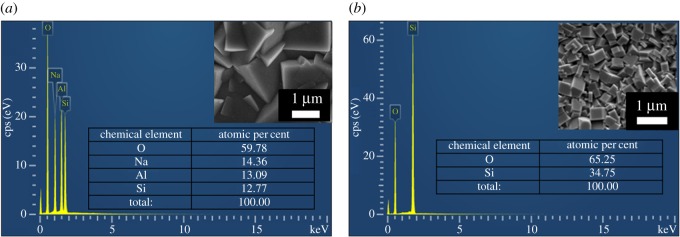
SEM–EDX chemical analysis on the surface of (*a*) a NaA membrane and (*b*) a silicalite-1 membrane on HNM supports.

The successful seeding on the HMM supports contributes to the continuous zeolite membrane formation after secondary growth, which needs less repetitive synthesis than those on α-Al_2_O_3_ supports. For the NaA zeolite membrane, after a twice secondary hydrothermal synthesis (scheme 1), the seed layer grew into a well-intergrown continuous LTA zeolite membrane with a thickness of approximately 5 µm (figure 5*a*,*b*). In the XRD pattern of the NaA zeolite membrane (figure 5*c* and electronic supplementary material), specific diffractive peaks could be attributed to the HNM support and LTA crystals. No impurity peaks of other crystals indicate that we have obtained pure LTA zeolite membrane. To further verify the purity of the LTA zeolite membrane, an SEM–EDS chemical analysis on the membrane surface (figure 6*a*) showed a silicon to aluminium ratio of 0.98 : 1 which is close to the standard NaA composition of **|**Na_91.7_**|**[Si_96_Al_96_O_384_], within the limiting error of the EDS instrument owing to membrane surface roughness. When an FNJ support was adapted for LTA membrane fabrication, a third-time hydrothermal synthesis was needed in order to get a continuous membrane, and the LTA membrane was out-of-flatness with a thickness greater than 10 µm (electronic supplementary material, figure S3) owing to the inhomogeneous seeding.

Similarly, a continuous silicalite-1 membrane composed of intergrown uniform silicalite-1 crystals of approximately 1.1 × 1.6 × 0.76 µm (figure 5*d*) with a uniform thickness of 3.3–3.8 µm (figure 5*e*) was fabricated by hydrothermal secondary growth of seed layer on HNM supports. The XRD pattern (figure 5*f* and electronic supplementary material) of the silicalite-1 membrane showed diffraction peaks that can be indexed to silicalite-1 zeolite crystalline structure and the HNM support. Although oxygen is difficult to quantify via EDS, the SEM–EDS chemical analysis of a selected surface of the calcined silicalite-1 membrane showed a composition ratio of silicon to oxygen of 1 : 1.88 in the silicalite-1 zeolite layer, close to the standard O/Si ratio of 2. No Al elements leached from the support (figure 6*b*), which also confirmed the integrity of these membranes. A continuous NaY zeolite membrane with a thickness less than 10 µm can also be prepared after a similar secondary growth process (figure 5*g*,*h*,*i* and electronic supplementary material). To be used as corroboration, *in situ* hydrothermal synthesis of LTA, MFI and FAU zeolite membranes without a seeding step yielded negative results, so that no continuous membranes could be formed on the HNM supports.

**Figure 7.**
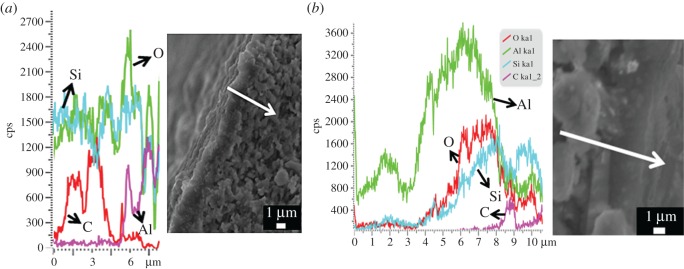
SEM–EDX linear scanning chemical analysis on the cross section of a silicalite-1 membrane on (*a*) an HNM support and (*b*) an α-Al_2_O_3_ support. The samples were uncalcined and carbon was used as a tracer element to show the membrane region.

To further prove that no Al leached from the support, we conducted an SEM–EDS linear scanning chemical analysis along the cross section of an uncalcined silicalite-1 membrane. Carbon was used as a tracer element to show the membrane region. The peaks of the carbon tracer appeared in the range of 0.6–4.9 µm of the abscissa, which can be assigned to the silicalite-1 domain top of the zeolite–halloysite interface. Again, no Al can be detected in this membrane layer region. The content of aluminium element increased beyond the interface after 5.1 µm, which can be attributed to the HNM supports (figure 7*a*).

As a comparison, aluminium element can be easily found in silicalite-1 membrane prepared on an α-Al_2_O_3_ support (figure 7*b*). The zeolite–alumina interface domain that contained carbon also showed a large amount of Al that monotonically decreased along the zeolite growth direction. As silicalite-1 is an all-silica zeolite, the Al must come from the α-Al_2_O_3_ support. Considering that silicalite-1 membrane is a widely used pure-silica zeolite membrane for organics removal from water [51], metallic elements leaching from a support can lower the hydrophobicity of the membrane in a devastating way [51,52] unless an additional intermediate protective layer is applied [53,54]. In this aspect, using HNM supports shows superiority to the commonly used α-Al_2_O_3_ supports. The Al leach-free phenomena may be explained by the microstructure of a natural halloysite nanotube (scheme 1), in which aluminium is buried in the inside structure, protected by a silica-like layer on the outer surface [55,56]. The silicon outer layer acts as a natural barrier to the Al transport from the support into the growing zeolite membrane. On the contrary, such a protective layer does not exist in sintered α-Al_2_O_3_ ceramics. Thus dissolution of Al from the support and penetration into the zeolite layer during the hydrothermal synthesis is inevitable under the alkaline synthesis condition [57]. This characteristic of no Al leaching from the HNM supports can be regarded as a congenital advantage for applications that need pure-silica zeolite layer-like separation membranes for organics removal [51] and also low-k materials for dielectric insulator applications [58].

### 3.3. Compatibility of aluminophosphate AlPO_4_-5 membrane on HNM supports with *in situ* hydrothermal synthesis

**Figure 8.**
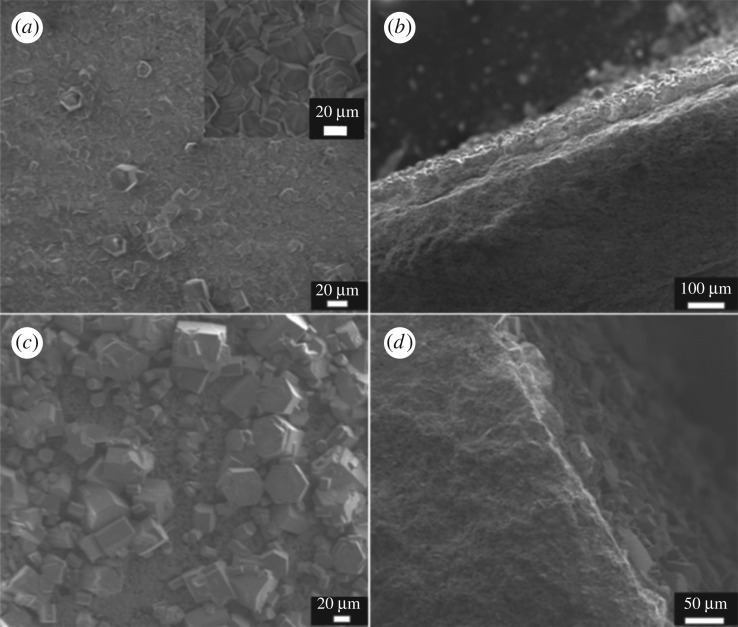
SEM images of (*a*) the top surface and (*b*) cross section of an AlPO_4_-5 membrane on HNM support, (*c*) the top surface and (*d*) cross section of an AlPO_4_-5 membrane on α-Al_2_O_3_ support.

Although the secondary growth method with a pre-seeding procedure on support has been demonstrated as an efficient strategy to decouple nucleation and crystal growth, so that both processes can be manipulated in a controlled way, *in situ* direct hydrothermal synthesis without a seeding procedure is far more time-saving and energy-saving. *In situ* hydrothermal synthesis of NaA, NaY and silicalite-1 membranes on HNM supports all failed. When using aluminophosphate AlPO_4_-5 as the zeolite layer, a continuous intergrown membrane was obtained. One-time *in situ* hydrothermal synthesis yielded a continuous intergrown AlPO_4_-5 membrane without macroscopic defect viewed under the scanning electron microscope (figure 8*a*). Big hexagonal columnar AlPO_4_-5 crystals with size approximately 21 × 35 µm could be found covering the entire HNM support. Cross-sectional images (figure 8*b*) showed a membrane thickness of approximately 45 µm, exhibiting no breakages and pinholes. The XRD pattern (electronic supplementary material) of the membrane detected characteristic diffraction peaks of AFI topology with no other redundant peaks, indicating a pure phase of AlPO_4_-5 (electronic supplementary material, figure S4). Only weak diffraction peaks of an HNM support can be found, probably because of the thick and integrated AlPO_4_-5 membrane. An SEM–EDS chemical analysis on the membrane surface (electronic supplementary material, figure S5) showed an aluminium-to-phosphorus ratio of 1 : 1, which is consistent with the standard AlPO_4_-5 zeolite composition. This AlPO_4_-5 membrane with a pore size of 0.73 × 0.73 nm could potentially be used in adsorption [59], separation applications [60] or as a catalytic membrane reactor [19,61].

When the HNM support was substituted by an α-Al_2_O_3_ support, no continuous membrane could be formed. Aluminophosphate AlPO_4_-5 crystals were discrete and sparsely distributed on the support. Most of the areas were bare without any zeolite crystals (figure 8*c*). The cross-sectional view of the membrane further illustrated that no continuous membrane has been synthesized on the α-Al_2_O_3_ support using this *in situ* hydrothermal method (figure 8*d*).

## 4. Conclusion

Self-standing ceramic nanofibre mats with high porosity, low tortuosity, uniform pore size distribution and high flux have been prepared by electrospinning and sintering. Halloysite clay with nanotube structure was mixed with PVP and then electrospun to form well-interlapped HNTs/PVP composite nanofibre mats. After sintering, novel ceramic nanofibre mats were fabricated with thickness up to 5 mm. The HNT-based nanofibre mat possessed a highly interconnected pore structure as well as omniphilicity. Therefore, it was particularly suitable to be a support for synthesizing molecular sieve membranes such as zeolite membranes. The HNT-based electrospun nanofibre mats could be better coated with zeolite seeds that were not easily detached by ultrasonic force, compared with α-Al_2_O_3_ supports. The natural protective layer of silica on the external surface of the nanotube prevented Al leaching from the support to the zeolite layer. Membranes of NaA, NaY and silicalite-1 zeolites by the secondary growth method, and AlPO_4_-5 membrane by the direct hydrothermal method have been successfully synthesized on the novel ceramic electrospun mats, whereas those on α-Al_2_O_3_ supports were either not as uniform, needed more repetitive synthesis or were not continuous. Separation experiments using these new composite zeolite membranes are currently underway in our laboratory.

## Supplementary Material

In the electronic supplementary material, Figure S1–S5 are included.

## Data accessibility

Our data are deposited at the Dryad Digital Repository: http://dx.doi.org/10.5061/dryad.cg385 [62].
