# Supplementary material for: Halloysite nanotube-based electrospun ceramic nanofibre mat: a novel support for zeolite membranes
Source: R Soc Open Sci. 2016 Dec 21;3(12):160552. doi: 10.1098/rsos.160552 (PMC5210680; doi:10.1098/rsos.160552)
Supplement: In the electronic supplementary material, Figure S1–S5 are included. [file rsos160552supp1.docx]

Supplementary Materials

Halloysite Nanotube-Based Electrospun Ceramic Nanofiber Mat: a Novel Support for Zeolite Membranes

*Zhuwen Chen,^†a^ Jiaying Zeng,^†b^ Dong Lv,^*b^ Jinqiang Gao,^a^ Jian Zhang,^a^ Shan Bai, ^a^ Ruili Li,^c^ Mei Hong,^*a^ Jingshen Wu^b^*

a, Guangdong Provincial Key Laboratory of Nano-Micro Materials Research, School of Chemical Biology & Biotechnology, Peking University Shenzhen Graduate School, Shenzhen 518055, China.

b, Department of Mechanical and Aerospace Engineering, The Hong Kong University of Science and Technology, Clear Water Bay, Hong Kong SAR, China.

c, Shenzhen Engineering Laboratory for Water Desalinization with Renewable Energy, School of Environment and Energy, Peking University Shenzhen Graduate School, Shenzhen 518055, China.


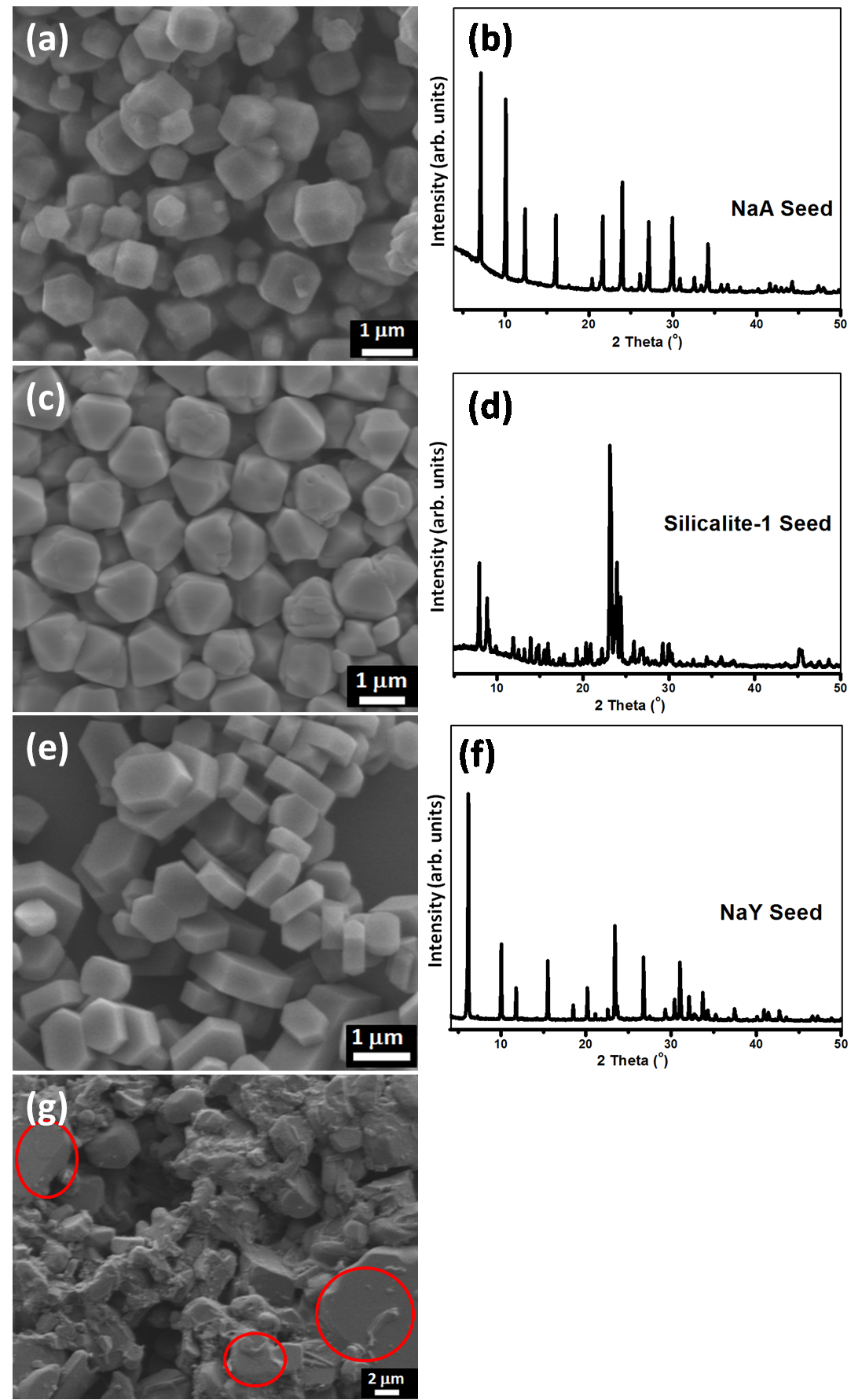


**Figure S1.** SEM images of (a) NaA (c) silicalite-1 (e) NaY seed crystals; XRD patterns (Data Availability) of (b) NaA (d) silicalite-1 (f) NaY seed crystals; (g) SEM image of the surface of an α-Al_2_O_3_ FNJ support, many surfaces of bulk α-Al_2_O_3_ grains are flat and smooth (marked out with red circles).


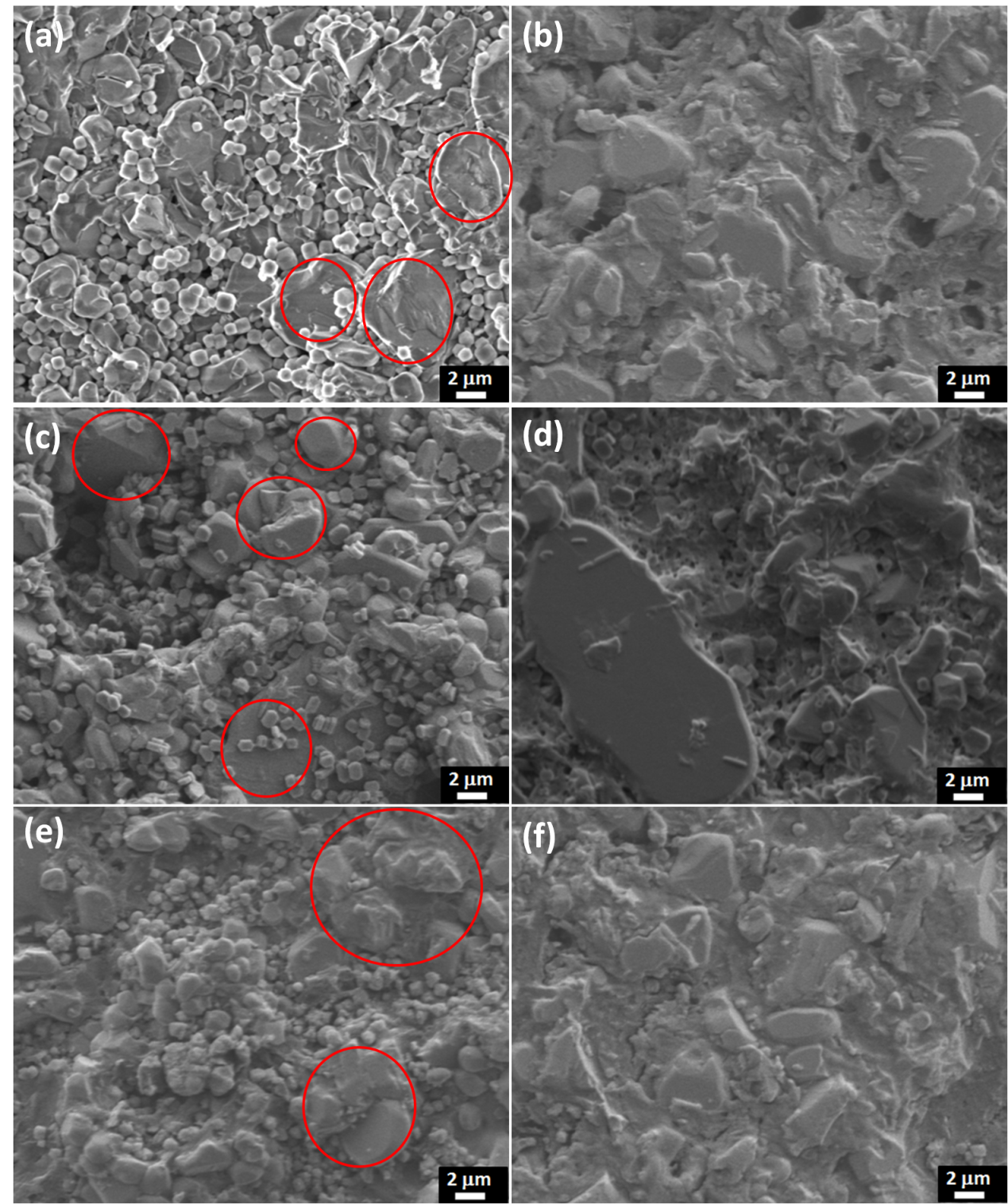


**Figure S2.** SEM microphotographs of (a) NaA (c) silicalite-1 (e) NaY crystals seeded FNJ support and corresponding images (b) NaA, (d) silicalite-1, (f) NaY seeded FNJ support after ultrasonic treatment for 2 min. A large portion of bulk α-Al_2_O_3_ grains are bare with no seeds attached (marked out with red circles).


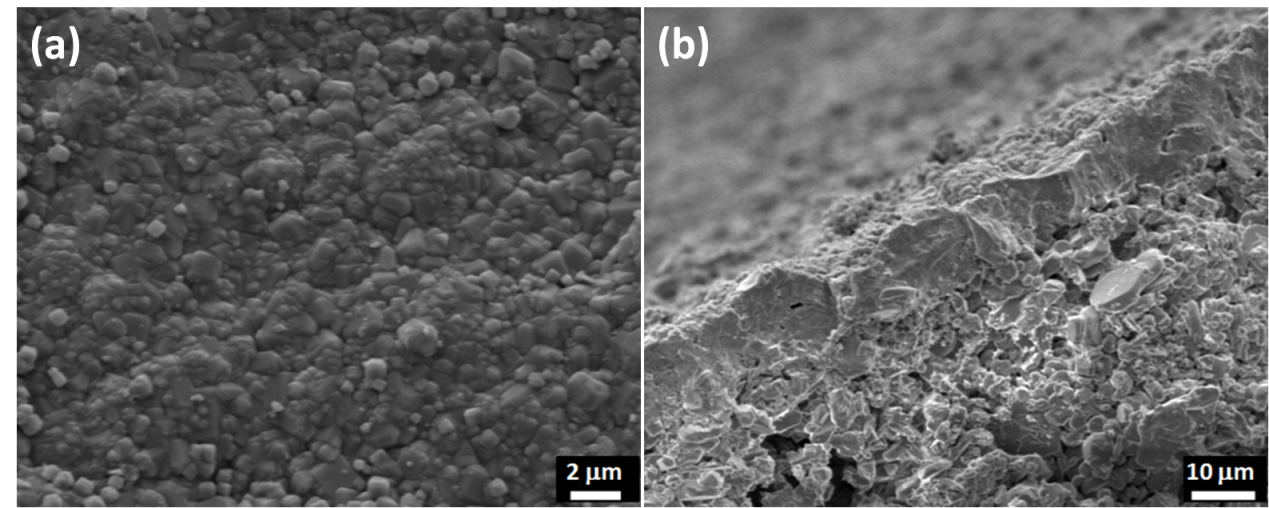


**Figure S3.** SEM microphotographs of (a) the surface (b) the cross section of a NaA zeolite membrane on a FNJ support. The membrane was prepared after a third time hydrothermal synthesis with a secondary growth method.


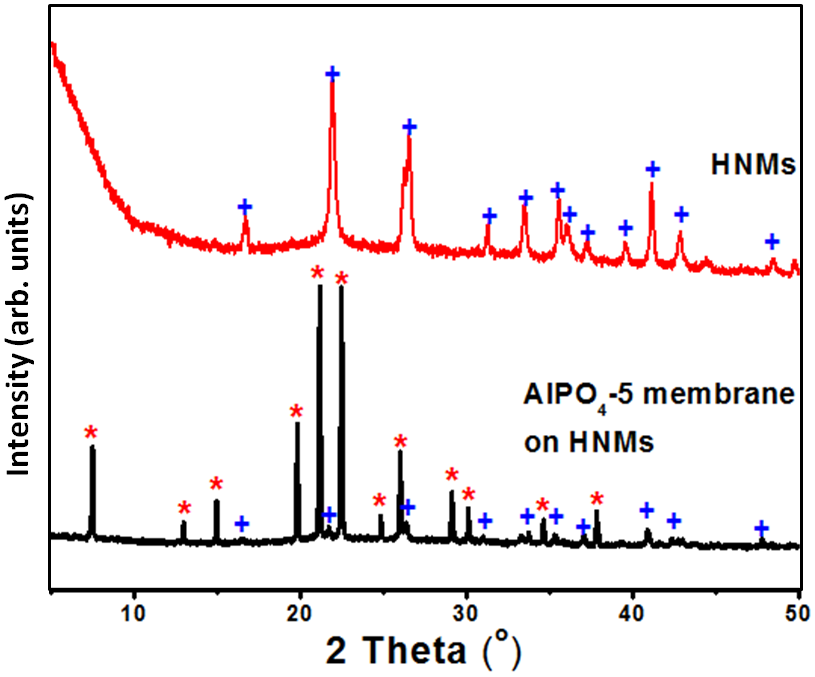


**Figure S4.** XRD patterns of a HNM support, and its supported AlPO_4_‑5 membrane, diffraction peaks of AlPO_4_‑5 zeolite crystals were marked with stars ＊, and diffraction peaks of HNMs were marked with crosses ＋.


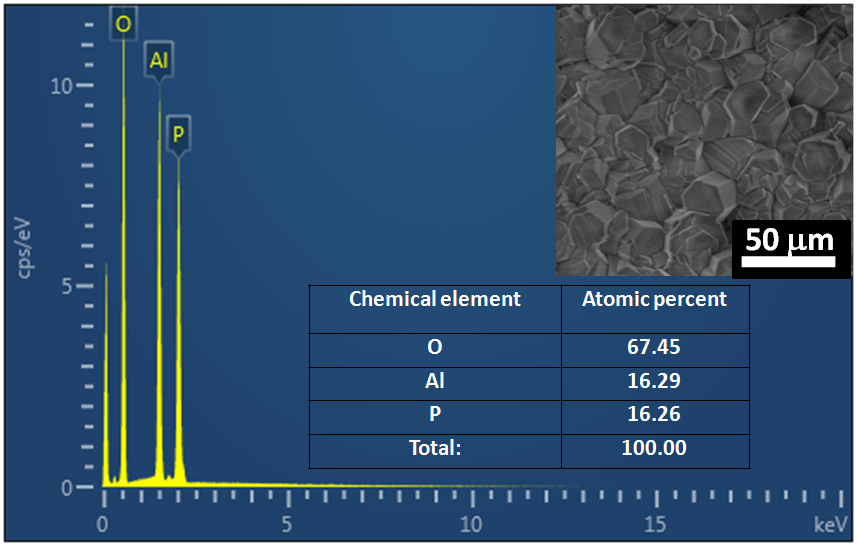


**Figure S5.** SEM-EDS chemical analysis on the surface of an AlPO_4_-5 membrane on a HNM support.
